# Disinfection efficiency test for contaminated surgical mask by using Ozone generator

**DOI:** 10.1186/s12879-022-07227-3

**Published:** 2022-03-07

**Authors:** Patcharaporn Tippayawat, Chalermchai Vongnarkpetch, Saitharn Papalee, Sukanya Srijampa, Thidarut Boonmars, Nonglak Meethong, Supranee Phanthanawiboon

**Affiliations:** 1 Center for Research and Development of Medical Diagnostic Laboratories (CMDL), Faculty of Associated Medical Sciences, Khon Kaen University, Khon Kaen, 40002 Thailand; 2 Department of Medical Technology, Faculty of Associated Medical Sciences, Khon Kaen University, Khon Kaen, 40002 Thailand; 3 Khon Kaen University Council Members, Khon Kaen University, Khon Kaen, 40002 Thailand; 4 Department of Microbiology, Faculty of Medicine, Khon Kaen University, Khon Kaen, 40002 Thailand; 5 Research and Diagnostic Center for Emerging Infectious Diseases (RCEID), Department of Microbiology, Faculty of Medicine, Khon Kaen University, Khon Kaen, 40002 Thailand; 6 Department of Parasitology, Faculty of Medicine, Khon Kaen University, Khon Kaen, 40002 Thailand; 7 Materials Science and Nanotechnology Program, Department of Physics, Faculty of Science, Khon Kaen University, Khon Kaen, 40002 Thailand

**Keywords:** Ozone, Viral disinfection, Bacterial disinfection, Fungal disinfection

## Abstract

**Background:**

Ozone (O_3_) is an effective disinfectant agent that leaves no harmful residues. Due to the global health crisis caused by the COVID-19 pandemic, surgical masks are in high demand, with some needing to be reused in certain regions. This study aims to evaluate the effects of O_3_ for pathogen disinfection on reused surgical masks in various conditions.

**Methods:**

O_3_ generators, a modified PZ 2–4 for Air (2000 mg O_3_/L) and a modified PZ 7 –2HO for Air (500 mg O_3_/L), were used together with 1.063 m^3^ (0.68 × 0.68 × 2.3 m) and 0.456 m^3^ (0.68 × 0.68 × 1.15 m) acrylic boxes as well as a room-sized 56 m^3^ (4 × 4 × 3.5 m) box to provide 3 conditions for the disinfection of masks contaminated with enveloped RNA virus (10^5^ FFU/mL), bacteria (10^3^ CFU/mL) and fungi (10^2^ spores/mL).

**Results:**

The virucidal effects were 82.99% and 81.70% after 15 min of treatment with 2000 mg/L O_3_ at 1.063 m^3^ and 500 mg/L O_3_ at 0.456 m^3^, respectively. The viral killing effect was increased over time and reached more than 95% after 2 h of incubation in both conditions. By using 2000 mg/L O_3_ in a 1.063 m^3^ box, the growth of bacteria and fungi was found to be completely inhibited on surgical masks after 30 min and 2 h of treatment, respectively. Using a lower-dose O_3_ generator at 500 mg O_3_/L in 0.456 m^3^ provided lower efficiency, although the difference was not significant. Using O_3_ at 2000 mg O_3_/L or 500 mg O_3_/L in a 56 m^3^ room is efficient for the disinfection of all pathogens on the surface of reused surgical masks.

**Conclusions:**

This study provided the conditions for using O_3_ (500–2000 mg/L) to reduce pathogens and disinfect contaminated surgical masks, which might be applied to reduce the inappropriate usage of reused surgical masks.

## Background

The current situation amid the novel coronavirus 2019 (COVID-19) pandemic has caused economic recession as well as mental health crises around the world. Citizens, especially health care workers, are at risk of infection. The virus spreads between people through small liquid particles due to coughing, sneezing, speaking, or even breathing. Infected secretions can remain in the air for several hours. The pathogen can survive on various surfaces for even longer periods depending on the type of material [1]. In addition to the coronavirus, bacteria or fungi can also be spread by exposure to air and environmental contaminants, including *Staphylococcus aureus* and *Pseudomonas aeruginosa*, which are common bacteria that cause infections in humans. Low immunity may cause infectious diseases in wound areas, surgical wounds, and lung infections [2, 3] from airborne transmission within hospitals or from other sources of contamination. These pathogens may also contaminate medical personnel. In addition, there are strains of fungi that can be transmitted through the air in the form of mycelium, mould, and spores such as *Aspergillus* spp., leading to hypersensitivities such as allergy and asthma [4, 5]. Masks have been recommended as a potential PPE to address the COVID-19 pandemic outbreak and other airborne pathogens. Reuse of a surgical mask is not recommended but has occurred during the recent high usage demands. Effective methods for the industrial disinfection of face masks include the use of hydrogen peroxide vapour, ultraviolet radiation, moist heat, dry heat, and ozone gas [6]. However, the optimal conditions for the disinfection of surgical masks for reuse are still understudied. Ozone is a molecule made up of 3 oxygen atoms (O_3_) with an unstable structure that has the ability to undergo oxidation reactions, making it toxic to microorganisms. Ozone is a gas that can spread over an area faster than regular liquid spraying. It undergoes oxidation with organic substances and can disinfect any inorganic substance in water and the air with a stronger sterilization effect on pseudoviruses, indicating that it can achieve coronavirus disinfection [7]. Several studies have shown that ozone can kill viruses on hard-to-reach surfaces, including the fabric structure of face masks, over a period of time [4] and that ozone kills 99% of airborne viruses in a period of 15 min [8]. The downside is that ozone can cause skin damage and respiratory irritation, which means it must be used with caution. However, it is highly unstable and has a short half-life and is thus easy to remove. In summary, ozone is a good candidate for surgical mask disinfection; however, the effectiveness of using ozone for disinfection depends on the concentration and time of treatment. Therefore, this study aims to investigate the efficacy of ozone against viral, bacterial, and fungal contamination on the surface of surgical masks. The results from this study will hopefully improve the understanding of the application of ozone in surgical mask disinfection.

## Methods

### O_3_ generator system

**Fig. 1:**
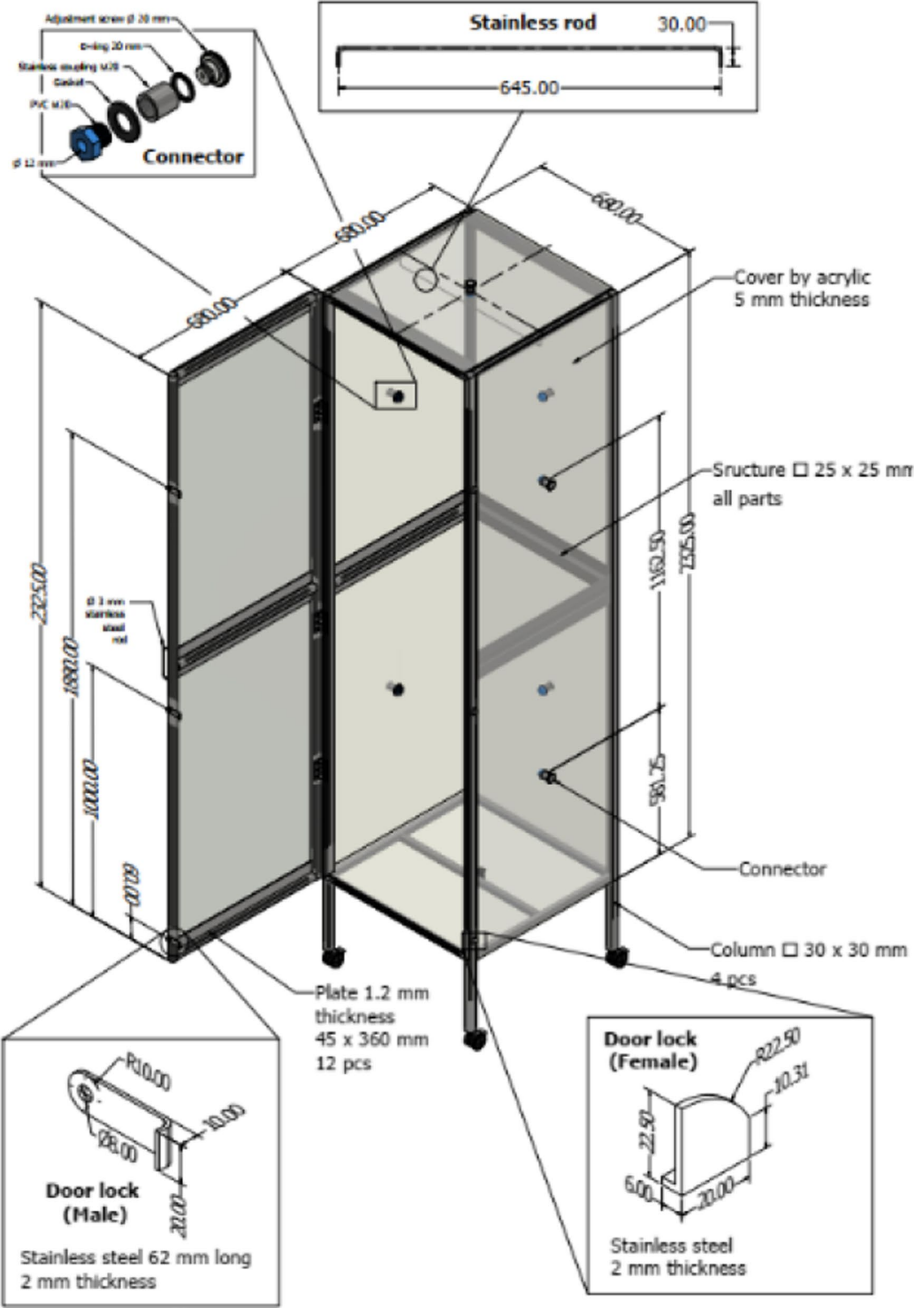
Acrylic box for connection to the O_3_ generator. Two pieces of 5 mm thick acrylic of size 0.68 × 1.15 (width × length) and 4 pieces of size 0.68 × 0.68 (width × length) were used to construct the box. Each side of the acrylic was designed to have a 25 × 25 mm connector for connection with the O_3_ generator and for opening to replace the O_3_ gas with O_2_. A manual lock was provided on the door side, and wheels were connected for easy movement

A modified PZ 2–4 for Air, which produced 2000 mg O_3_/L, and a modified PZ 7 –2HO for Air, which produced 500 mg O_3_/L, were used together with acrylic boxes. A box sized 0.68 × 0.68 × 2.3 m (1.063 m^3^) was made of 5 mm thick acrylic with a connector on each side of the box to be easily used with the modified PZ 2–4 for Air O_3_ generator and to be opened for decontamination of the O_3_ after completing the experiment by replacing the O_3_ with O_2_, as shown in Fig. 1. A half-size box at 0.456 m^3^ (0.68 × 0.68 × 1.15 m) capacity was constructed the same way (data not shown) for use with a smaller O_3_ generator, the modified PZ 7 –2HO for Air. Experimentation was performed immediately after gaseous O_3_ from the O_3_ generator was introduced into the box until the O_3_ metre reached 10 ppt. Disinfection of a contaminated mask in a room was performed in a room-sized 56 m^3^ (4 × 4 × 3.5 m) chamber at room temperature and humidity.

### Viral preparation

Dengue virus, which is a representative RNA enveloped virus, was propagated in the C6/36 mosquito cell line in a T75 flask at a multiplicity of infection (MOI) of 0.1 [9]. The inoculated cells were incubated at 28 °C without CO_2_ for 7 days before removal of the supernatant containing new progeny viruses. Infectious particles in the collected supernatant were tested by the focus-forming assay (FFA) followed by the indirect immunofluorescent assay (IFA).

### Virus titration (focus forming assay)

Viral infectivity was evaluated and represented as focus forming units per millilitre (FFU/mL) by the focus forming assay [10]. Briefly, monolayer Vero cells in Dulbecco’s modified Eagle’s medium (DMEM) (Gibco, USA) supplemented with 10% foetal bovine serum (FBS) were prepared in a sterile 96-well plate one day before the experiment and incubated at 37 °C with 5% CO_2_. The supernatant containing the virus was diluted to 1:10^7^ by DMEM on ice before being introduced to 50 µl of cells. Inoculated cells were incubated for 2 h with shaking every 30 min to allow viral infection. A sticky reagent (2% carboxymethyl cellulose (CMC) in DMEM) was added on top to limit viral spreading. Infected cells were incubated at 37 °C with 5% CO_2_ for 3 days before fixation and permeabilization by 4% formaldehyde in phosphate-buffered saline (PBS) (Sigma Aldrich, USA) and 0.1% Triton X-100 in PBS (Sigma Aldrich, USA). Fixed cells were primed with a primary antibody specific to the dengue virus followed by a secondary antibody labelled with Alexa488 for visualization under a fluorescence microscope. The number of foci was counted and calculated to determine the number of focus forming units (FFU)/mL [11].

### Efficiency of ozone efficiency for viral disinfection on contaminated surgical masks

The number of mask-contaminating pathogens was identified by a standard pathogen counting technique before and after ozone treatment under the various conditions. The variables included the concentration of ozone, container size, and time of exposure. To evaluate the viral disinfection efficiency of ozone under various conditions, the optimal concentration of the virus was prepared for the test. A virus concentration of 10^5^ FFU/mL was prepared on ice, and 100 µl (10,000 FFU) was introduced to a sterile surgical mask sized 1 cm^2^ before placing it in a sterile petri dish. A dish with a contaminated mask was placed in 3 disinfectant conditions: 0.53 m^3^ with O_3_ 500 mg/L, 1.6 m^3^ with O_3_ 2000 mg/L, 56 m^3^ with O_3_ 500 and 2000 mg/L, and with the cover open before running the machine. Time was counted from immediately after 10 parts per trillion (ppt) were measured by the O_3_ measurement machine (Prozone, Thailand). The contaminated mask was collected from each disinfectant condition after 0 min, 15 min, 30 min, 1 h and 2 h of O_3_ treatment at room temperature in August in Thailand. For the decontamination of the mask at room temperature, 4 h of O_3_ treatment was added. The contaminated mask was submerged in 200 µl of sterile DMEM to transfer the virus into the culture medium. The culture medium was subjected to FFA for comparison to the control virus at the starting point.

### Efficiency of ozone for bacterial and fungal disinfection on contaminated surgical masks

To determine the antibacterial and antifungal activity of ozone, gram-positive and gram-negative bacteria, namely, *Staphylococcus aureus* (*S. aureus*) ATCC29213, *Pseudomonas aeruginosa* (*P. aeruginosa*) ATCC27803, and the fungus *Aspergillus* spp. were used as representative pathogens. The bacteria were subcultured in nutrient broth (NB) and incubated at 37 °C overnight. Subsequently, the organisms were washed by centrifugation and resuspended in 0.9% sodium chloride (normal saline solution), and the concentration was measured spectrophotometrically at 600 nm. Then, the bacteria were adjusted to the desired concentrations with normal saline solution.

For fungal preparation, *Aspergillus* spp*.* was cultured on Sabouraud dextrose agar (SDA) and incubated at 25 °C for 3 days. The mould spores were transferred to 0.1% peptone water by using a needle. Then, the spores were counted with a haemocytometer and adjusted to the required concentration with normal saline solution for the experiment.

The bacterial concentration of 10^3^ colony forming units (CFU)/mL and the *Aspergillus* spp. concentration of 10^2^ spores/mL were separately dropped onto a sterile 1 cm^2^ piece of surgical mask and placed in a sterile petri dish. The dishes were placed in a small box (0.53 m^3^; 500 mg/L) and a large box (1.6 m^3^; 2000 mg/L), and ozone was released through the channel at the cabinet base into the tank until the ozone density reached 10 ppt. The contaminated masks were collected from each disinfectant condition after 0 min, 15 min, 30 min, 1 h, and 2 h of O_3_ treatment. The fungus-contaminated masks were placed on the SDA. The bacteria-contaminated masks were cultured in sterile nutrient broth and placed on a Mueller–Hinton agar (MHA) surface. Then, the samples were incubated at 37 °C overnight to check the sterility of the contaminated masks [12, 13].

## Results

### Viral disinfection

**Fig. 2:**
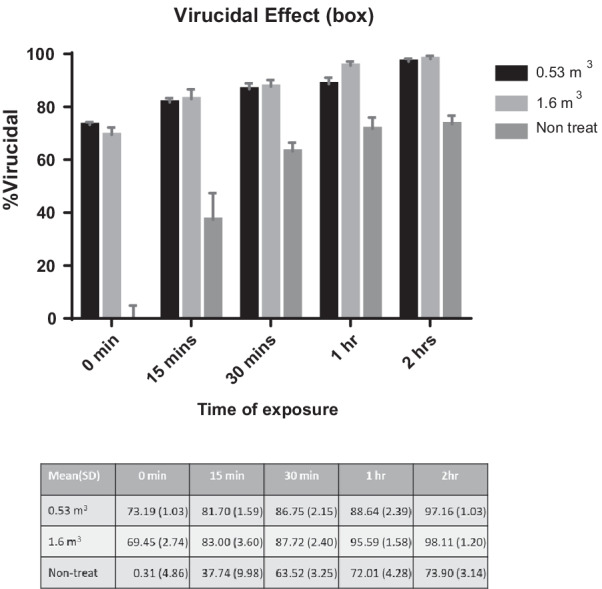
Percent virucidal effect of ozone treatment at different times of exposure. The virucidal effects of ozone were determined in a 0.53 m^3^ box (black bars) and a 1.6 m^3^ box (light grey bars) after 0 min, 15 min, 30 min, 1 h and 2 h of treatment. The dark grey bars show the percentage (%) death of the virus in a control tube without O_3_ treatment. The data represent the mean and SD of the ozone killing effect, and the value of each is also shown in the table under the graph

**Fig. 3:**
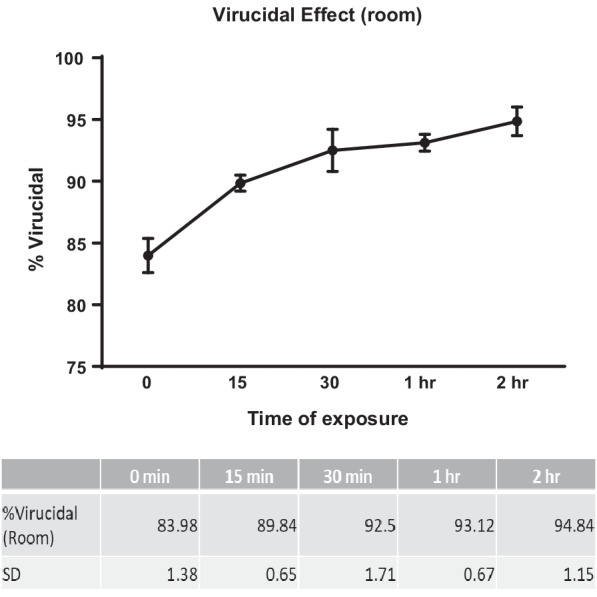
Percent virucidal effect of ozone treatment by using O_3_ 2000 mg/L and 500 mg/L in a 56 m^3^ room after 0 min, 15 min, 30 min, 1 h and 2 h of treatment. The data represent the mean and SD of the ozone killing effect, and the values are shown in the table under the graph

At O_3_ concentrations of 2000 mg/L in a 1.6 m^3^ box and 500 mg/L in a 0.53 m^3^ box, the infectious viral particles were inhibited by 82.99% and 81.70% after 15 min of treatment compared to the non-O_3_-treated virus control. The virucidal effect increased in a time-dependent manner in both conditions: 87.71% and 86.75% at 30 min, 95.59% and 88.64% at 1 h and 98.11% and 97.16% at 2 h of incubation in 1.6 m^3^ and 0.53 m^3^ boxes, respectively (Fig. 2). Compared to the virus control, the killing effect was also increased due to the fragile character of the virus at room temperature. To completely eliminate the virus, 2000 mg/L and 500 mg/L treatment for more than 2 h would be required. Regarding the killing effect of the virus in the room-sized space of 56 m^3^ with O_3_ concentrations of 2000 mg/L and 500 mg/L, the amount of the virus was reduced by treatment with O_3_ from the beginning of treatment (83.98%), and the virucidal effect increased to 89.84%, 92.5%, 93.12% and 94.84% after 15 min, 30 min, 1 h and 2 h of incubation (Fig. 3). The effect of O_3_ in decontamination depended on the concentration and the treatment time.

### Bacteria and fungus disinfection in a closed-system ozone incubator

**Fig. 4:**
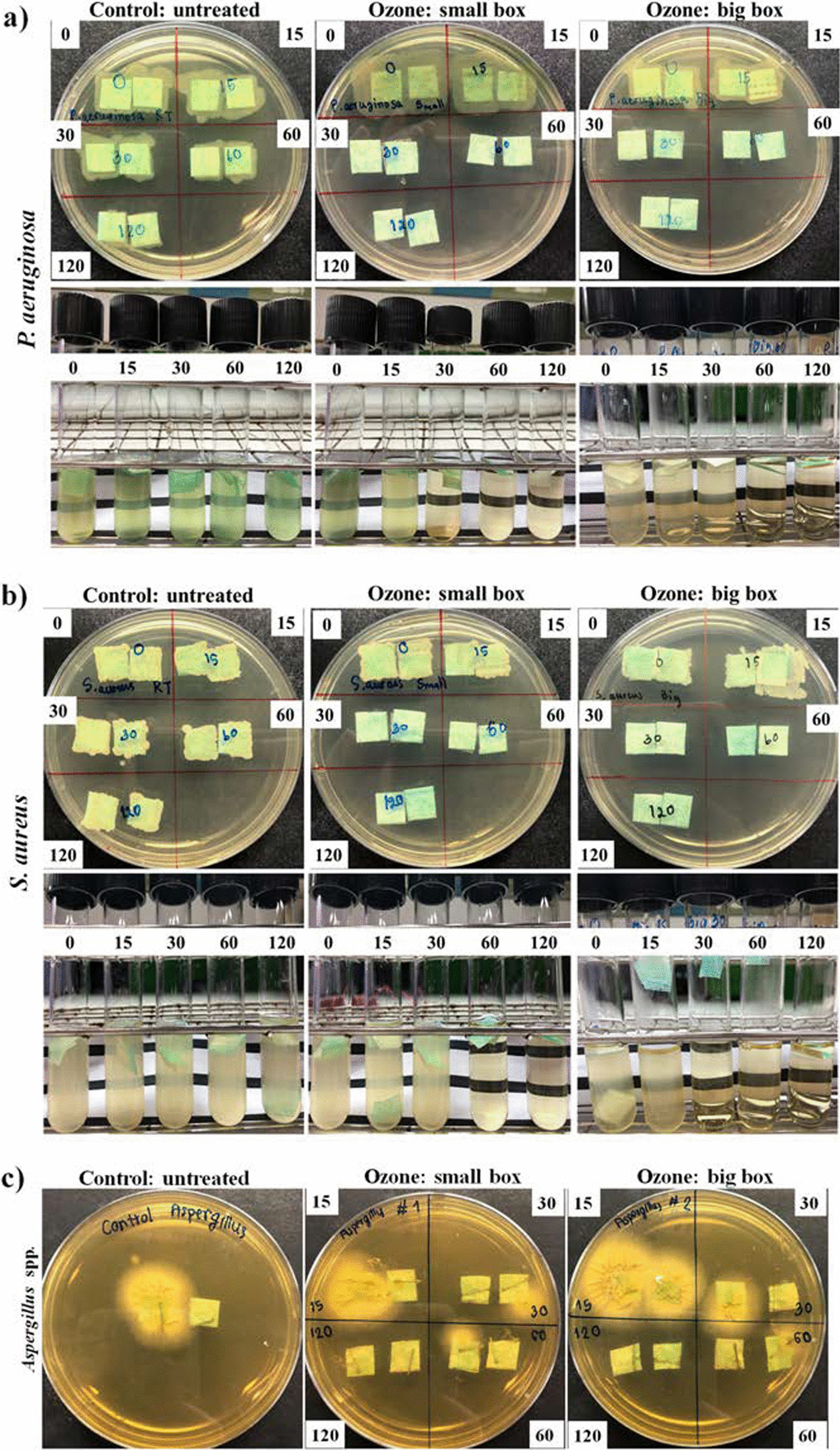
Potential of O_3_ to kill **a** *P. aeruginosa* **b** *S. aureus* and **c** *Aspergillus* spp. at different intervals (0 min, 15 min, 30 min, 1 h, and 2 h) in the small box (0.53 m^3^) and large box (1.6 m^3^) compared to the untreated control

**Fig. 5:**
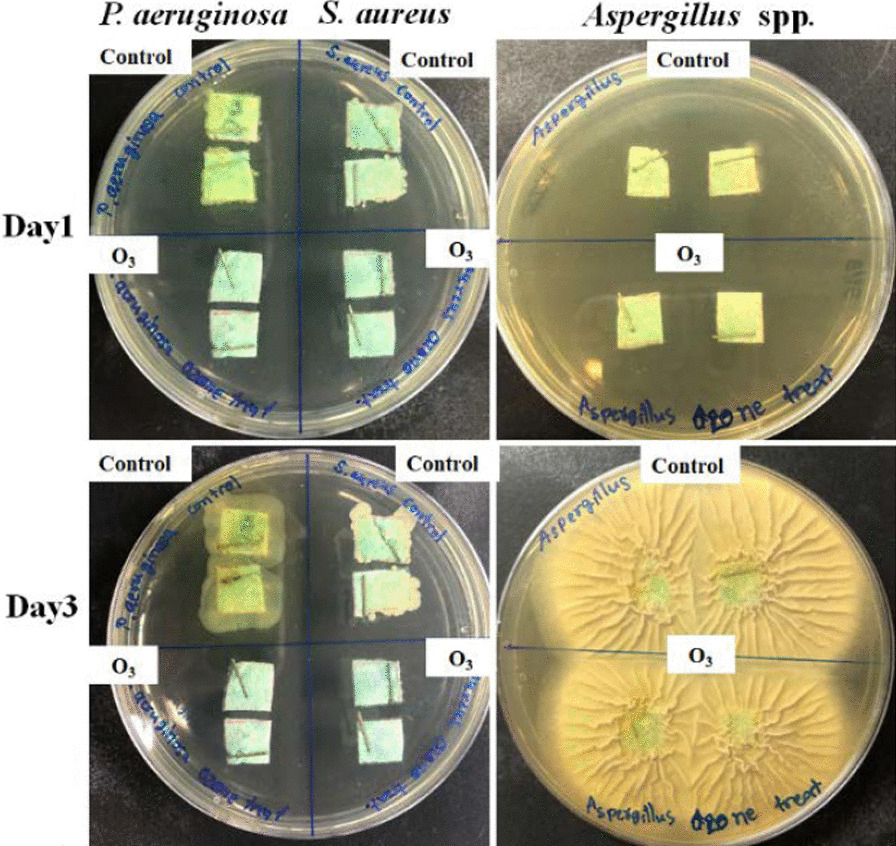
Ozone killing action against *P. aeruginosa* and *S. aureus* and *Aspergillus* spp. after 4 h in the room compared to the control (untreated)

The *P. aeruginosa, S. aureus* and *Aspergillus* spp. disinfection capability of ozone was tested in a closed-system ozone incubator. The results showed that ozone treatment in small- and large-box conditions could completely inhibit the growth of 10^3^ CFU/mL *P. aeruginosa* and *S. aureus* on the mask after 60 and 30 min of treatment, respectively, as shown in Fig. 4. In addition, *Aspergillus* spp. at a concentration of 10^2^ spores/mL was eliminated within 120 min. In addition, the results of the chamber sterilization experiment showed that bacterial microorganisms were disinfected within 4 h. However, fungal microorganisms were only partially disinfected (Fig. 5).

## Discussion

Wearing a mask is one of the best practices to avoid COVID-19 spread and infection, as recommended by the World Health Organization (WHO. It could also be used for other pandemic infections. Several methods, such as high temperature, UV, ozone, and hydrogen peroxide, have been applied for the reuse, disinfection, and sterilization of disposable masks to avoid a lack of usage in crises and for safety. Each type of mask may require a different method depending on the material used in construction.

Here, we propose the application of O_3_ in a certain sized container for the reduction and elimination of bacteria and viruses on surgical mask material. A surgical mask is a widely used tool for medical staff in hospitals as well as ordinary people. However, studies concerning the reuse, disinfection, and sterilization of surgical masks are rare compared to those for N95 or filtering facepiece (FFP) respirators [14].

Our results indicated the effectiveness of low-dose O_3_ (2000 mg/L: 1.02 ppm and 500 mg/L: 0.26 ppm) in decontaminating surgical masks by reducing the amount and inhibiting the growth of viruses, bacteria, and fungi after 15 min, 30 min, and 2 h of treatment with O_3_ produced from the modified PZ 2–4, which generates 2000 mg O_3_/L in a 0.53 m^3^ box. The results are similar to the findings of previous studies in terms of the efficacy of O_3_ in killing pathogens on surfaces. Dennis et al. found that gaseous O_3_ inactivated SARS-CoV-2. They also proposed a practical recommendation to implement a simple O_3_ disinfection box for FFP respirators with 10–20 ppm O_3_ for at least 10 min. The literature suggests that ozone attacks capsid proteins in nonenveloped viruses and most readily attacks enveloped viruses [15, 16]. The effectiveness of O_3_ for killing viruses depends on the relative humidity, temperature, and type of virus, as shown in Dubuis et al. 2020, who reported that a higher effect of low-dose O_3_ exposure (0.23–1.23 ppm) for the inactivation of norovirus was found at 85% relative humidity (RH) for 40 min norovirus, while 20% RH for 10 min gave the same result for bacteriophages. These results suggested that high RH should be used together with O_3_ to obtain a powerful disinfectant for airborne viruses, which could be implemented inside hospital rooms that are ventilated naturally. However, this study was performed under temperature and humidity conditions in August in Thailand without measuring the exact temperature and RH, although the average temperature was 28 °C and the average relative humidity was 83.2% according to the August 2020 agrometeorological report by the meteorological department [17].

Gram-negative bacteria and fungi require more time for decontamination. There are many reports of O_3_ lowering the number of bacteria, viruses, and bacterial spores on the surfaces of materials, including figs, fabrics, and plastics, at a relatively low concentration of 1–25 ppm in an average time of 1–4 h [18, 19]. These results link to this study and the experiment of *P. aeruginosa* and *S. aureus* closed-system disinfection in a closed system, which showed that bacteria at a concentration of 10^3^ CFU/mL were eliminated within 30 min, and chamber sterilization was achieved within 4 h. Moreover, this experiment successfully achieved the fungal inactivation of *Aspergillus* spp. by ozone in a closed-system ozone incubator within 120 min. This can be related to previous studies that showed similar results for fungal inactivation. Wood et al. reported on the inactivation of spores of *Bacillus anthracis* and *Bacillus subtilis* on building materials by O_3_ [20]. O_3_ can diffuse through the cell membrane, and attacking glycoproteins and glycolipids in the cell membrane results in the rupture of pathogen cells. Moreover, O_3_ attacks the sulfhydryl groups of certain enzymes, resulting in disruption of normal cellular enzymatic activity and loss of function. Ozone also attacks the purine and pyrimidine bases of nucleic acids, damaging DNA [21, 22]. The advantages of ozone gas are that it reaches shadows and crevices in the process of disinfection, unlike ultraviolet radiation which has a short half-life in an airflow environment. The immediately dangerous to life or health concentration (IDLH) of ozone is 5 ppm for humans. Exposure to 50 ppm for 60 min will probably be fatal to humans [23]. Therefore, a low dose in a closed system should be used to avoid direct contact. However, O_3_ gas can be exchanged quickly by O_2,_ and the odour of O_3_ is detectable by many people at low concentrations of 0.1 ppm in air in a home environment with air changes per hour varying between 5 and 8 ACH. Ozone has a half-life as short as 30 min [24], and the reaction proceeds faster at higher temperatures (Earth Science FAQ in the picture). Our experiment used a generator machine that produced 2000 mg/L in a 0.53 m^3^ box.

This study also supported previous studies showing that treatment with ozone causes very low degradation to fibrous structures or the fit of surgical masks. This is unlike other decontamination procedures, such as UV treatment, which enables reuse a limited number of times because of negative side effects, including deformation of the elastic, the accumulation of humidity, and destruction of the fibrous material. This suggested that O_3_ treatment could maintain the filtration capacity of a mask for reuse more than 30 times [25].

Only 2 sizes of container and 2 concentrations of O_3_ were used in this study. The temperature and humidity during the experiment were not fixed, which may affect the disinfectant efficiency of ozone, and the filtration capacity of the surgical mask was not determined.

## Conclusions

In conclusion, the results of this study supported the possibility of using O_3_ as an effective procedure for the decontamination of reused surgical masks at a dose of 2000 mg/L O_3_ in a 0.53 m^3^ box for 2 h, which could decontaminate surgical masks for reuse by reducing and eliminating the level of pathogens, including bacteria, viruses, and fungi. Longer exposure times lead to greater viral inactivation. Nevertheless, risks for user safety and health remain. Therefore, ozone should be used and handled properly.

## Data Availability

The data that support the findings of this study are available from the corresponding author upon reasonable request.

## References

[1] Jayaweera M, Perera H, Gunawardana B, Manatunge J (2020). Transmission of COVID-19 virus by droplets and aerosols: a critical review on the unresolved dichotomy. Environ Res.

[2] Brazova J, Sediva A, Pospisilova D, Vavrova V, Pohunek P, Macek M, Bartunkova J, Lauschmann H (2005). Differential cytokine profile in children with cystic fibrosis. Clin Immunol.

[3] Chuang CH, Wang YH, Chang HJ, Chen HL, Huang YC, Lin TY, Ozer EA, Allen JP, Hauser AR, Chiu CH (2014). Shanghai fever: a distinct *Pseudomonas aeruginosa* enteric disease. Gut.

[4] Lee J, Bong C, Lim W, Bae PK, Abafogi AT, Baek SH, Shin YB, Bak MS, Park S (2021). Fast and easy disinfection of coronavirus-contaminated face masks using ozone gas produced by a dielectric barrier discharge plasma generator. Environ Sci Tech Lett.

[5] Chopra V, Jain H, Goel AD, Chopra S, Chahal AS, Garg N, Mittal V (2017). Correlation of aspergillus skin hypersensitivity with the duration and severity of asthma. Monaldi Arch Chest Dis.

[6] Rubio-Romero JC, Pardo-Ferreira MDC, Torrecilla-Garcia JA, Calero-Castro S (2020). Disposable masks: Disinfection and sterilization for reuse, and non-certified manufacturing, in the face of shortages during the COVID-19 pandemic. Saf Sci.

[7] Zucker I, Lester Y, Alter J, Werbner M, Yecheskel Y, Gal-Tanamy M, Dessau M. Pseudoviruses for the assessment of coronavirus disinfection by ozone. Environ Chem Lett 2021:1–7. 10.1007/s10311-020-01160-0 PMC7805571 33462542

[8] Tseng CC, Li CS (2006). Ozone for inactivation of aerosolized bacteriophages. Aerosol Sci Tech.

[9] Hitakarun A, Ramphan S, Wikan N, Smith DR (2020). Analysis of the virus propagation profile of 14 dengue virus isolates in Aedes albopictus C6/36 cells. BMC Res Notes.

[10] Fujita N, Tamura M, Hotta S (1975). Dengue virus plaque formation on microplate cultures and its application to virus neutralization (38564). Proc Soc Exp Biol Med.

[11] Payne AF, Binduga-Gajewska I, Kauffman EB, Kramer LD (2006). Quantitation of flaviviruses by fluorescent focus assay. J Virol Methods.

[12] Guideline for Disinfection and Sterilization in Healthcare Facilities, 2008. https://www.cdc.gov/infectioncontrol/guidelines/disinfection/.

[13] Eissa M, Naby M, Beshir M (2014). Bacterial vs fungal spore resistance to peroxygen biocide on inanimate surfaces. Bull Faculty Pharmacy, Cairo University.

[14] Standard 62.2-2019—American Society of Heating, Refrigerating and Air-Conditioning Engineers. https://ashrae.iwrapper.com/ASHRAE_PREVIEW_ONLY_STANDARDS/STD_62.2_2019.

[15] Tseng C, Li C (2008). Inactivation of surface viruses by gaseous ozone. J Environ Health.

[16] Rojas-Valencia MN. Research on ozone application as disinfectant and action mechanisms on wastewater microorganisms. In: 2012; 2012.

[17] Agrometeorological Report August 2020. http://www.arcims.tmd.go.th/DailyDATA/Agroreport/รายงานอุตุนิยมวิทยาเกษตรเดือนสิงหาคม2563.pdf.

[18] Sharma M, Hudson JB (2008). Ozone gas is an effective and practical antibacterial agent. Am J Infect Control.

[19] Akbas MY, Ozdemir M (2008). Effect of gaseous ozone on microbial inactivation and sensory of flaked red peppers. Int J Food Sci Technol.

[20] Wood JP, Wendling M, Richter W, Rogers J (2020). The use of ozone gas for the inactivation of Bacillus anthracis and Bacillus subtilis spores on building materials. PLoS ONE.

[21] Wagner JR, Madugundu GS, Cadet J (2021). Ozone-induced DNA damage: a Pandora’s box of oxidatively modified DNA bases. Chem Res Toxicol.

[22] Cataldo F (2006). DNA degradation with ozone. Int J Biol Macromol.

[23] King ME (1963). Toxicity of ozone. V. Factors affecting acute toxicity. Ind Med Surg.

[24] Moore J, Maier D, Ileleji K (2013). Half-life time of ozone as a function of air movement and conditions in a sealed container. J Stored Prod Res.

[25] Dennis R, Pourdeyhimi B, Cashion A, Emanuel S, Hubbard D. Durability of disposable N95 mask material when exposed to improvised ozone gas disinfection. J Sci Med. 2020; 2.

